# Monitoring School Absenteeism for Influenza-Like Illness Surveillance: Systematic Review and Meta-analysis

**DOI:** 10.2196/41329

**Published:** 2023-01-11

**Authors:** Tim K Tsang, Xiaotong Huang, Yiyang Guo, Eric H Y Lau, Benjamin J Cowling, Dennis K M Ip

**Affiliations:** 1 WHO Collaborating Centre for Infectious Disease Epidemiology and Control, School of Public Health Li Ka Shing Faculty of Medicine The University of Hong Kong Hong Kong China (Hong Kong); 2 Laboratory of Data Discovery for Health Limited Hong Kong Science and Technology Park Hong Kong China (Hong Kong)

**Keywords:** influenza, surveillance, school absenteeism, monitoring, school attendance, influenza-like illness, correlation, trend, pattern, predict, prediction, influenza activity, infection, surveillance tolls

## Abstract

**Background:**

Influenza causes considerable disease burden each year, particularly in children. Monitoring school absenteeism has long been proposed as a surveillance tool of influenza activity in the community, but the practice of school absenteeism could be varying, and the potential of such usage remains unclear.

**Objective:**

The aim of this paper is to determine the potential of monitoring school absenteeism as a surveillance tool of influenza.

**Methods:**

We conducted a systematic review of the published literature on the relationship between school absenteeism and influenza activity in the community. We categorized the types of school absenteeism and influenza activity in the community to determine the correlation between these data streams. We also extracted this correlation with different lags in community surveillance to determine the potential of using school absenteeism as a leading indicator of influenza activity.

**Results:**

Among the 35 identified studies, 22 (63%), 12 (34%), and 8 (23%) studies monitored all-cause, illness-specific, and influenza-like illness (ILI)–specific absents, respectively, and 16 (46%) used quantitative approaches and provided 33 estimates on the temporal correlation between school absenteeism and influenza activity in the community. The pooled estimate of correlation between school absenteeism and community surveillance without lag, with 1-week lag, and with 2-week lag were 0.44 (95% CI 0.34, 0.53), 0.29 (95% CI 0.15, 0.42), and 0.21 (95% CI 0.11, 0.31), respectively. The correlation between influenza activity in the community and ILI-specific absenteeism was higher than that between influenza activity in community all-cause absenteeism. Among the 19 studies that used qualitative approaches, 15 (79%) concluded that school absenteeism was in concordance with, coincided with, or was associated with community surveillance. Of the 35 identified studies, only 6 (17%) attempted to predict influenza activity in the community from school absenteeism surveillance.

**Conclusions:**

There was a moderate correlation between school absenteeism and influenza activity in the community. The smaller correlation between school absenteeism and community surveillance with lag, compared to without lag, suggested that careful application was required to use school absenteeism as a leading indicator of influenza epidemics. ILI-specific absenteeism could monitor influenza activity more closely, but the required resource or school participation willingness may require careful consideration to weight against the associated costs. Further development is required to use and optimize the use of school absenteeism to predict influenza activity. In particular, the potential of using more advanced statistical models and validation of the predictions should be explored.

## Introduction

Influenza virus causes substantial morbidity and mortality in humans each year on average [1,2]. Influenza activity and hence infections decreased due to public health and social measures of COVID-19 [3]. However, rebound of influenza virus activity is expected [4,5], given the relaxation of public health and social measures [6,7] and low population immunity of influenza [8]. Therefore, monitoring influenza activity is important in the post–COVID-19 era. Common modes of surveillance for influenza activity include the following: (1) sentinel surveillance, in which the consultation rates or the number of influenza-like illness (ILI) in outpatient and private medical practitioner clinics is recorded and (2) laboratory surveillance, in which respiratory specimens are collected and the proportion of positive tests of the influenza virus are recorded.

Children are believed to be a major driver of influenza virus transmission since they have more frequent person-to-person close contacts with low preexisting immunity [9-11]. Therefore, school is a high-risk setting for the transmission of influenza and other respiratory viruses. Preventing transmission in schools could block transmissions to family members and further reduce community epidemics [12]. School surveillance offers an opportunity for the early detection of these viruses [13]. In some regions, student absenteeism surveillance could be in real time, and hence it could have minimal reporting delay, with relative low cost [14,15].

Some studies explore the potential use of school absenteeism to monitoring the influenza activity in the community. In some regions, school absenteeism has been integrated as a part of routine disease surveillance, particularly for respiratory virus infections [16-21], although there are also usages for other diseases [22,23]. However, the component of school absenteeism surveillance could be different, such as the use of all-cause and illness-specific absenteeism. Moreover, their performances are rarely assessed. Therefore, validating and optimizing the performance of the use of school absenteeism on the surveillance of influenza activity would be critical.

In this paper, we conducted a systematic review to collect information on studies that described both school absenteeism surveillance and community surveillance. We summarized the types of school absenteeism surveillance and community surveillance methods to assess the relationship between these 2 surveillances, and the potential use of school absenteeism surveillance to predict influenza activity in the community.

## Methods

### Definition of School Absenteeism Surveillance and Community Surveillance

School absenteeism surveillance was defined as the time series recording the absent rate per day or week in a school. Absenteeism could be all-cause, specific to ILI, or specific to respiratory illness but not limited to ILI.

Community surveillance was defined as the time series monitoring the influenza activity in a region. There were the following two main types of surveillance: (1) ILI rate or count per day or week and (2) the proportion of laboratory specimens testing positive for influenza virus.

### Search Strategy and Selection Criteria

This systematic review was conducted following the PRISMA (Preferred Reporting Items for Systematic Review and Meta-analysis) statement [24]. A standardized search was done in PubMed, Embase, and Web of Science, using the search term “((school AND (absent OR absence OR absenteeism) AND (flu OR influenza))”. The search was done on May 12, 2022, with no language restrictions. Additional relevant articles from the reference sections were also reviewed.

Two authors (XH and YG) independently screened the titles and extracted data from the included studies. Disagreements were resolved by consensus with a third author (TKT). Studies identified from different databases were deduplicated.

Eligible articles were those reporting daily or weekly student absenteeism data and community ILI surveillance in the same region. There was no restriction on the methods used for finding the relationship between school absenteeism and influenza surveillance in the community. Studies without both school absenteeism and community surveillance data were excluded. Articles that met the following were also excluded: (1) the study summarized the findings published elsewhere; (2) the study used data generated from simulation or prediction; (3) the study analyzed the combination of data from studies published elsewhere; or (4) the full text was not available.

Data were extracted from included studies using a standard form, with the following information in following 3 major components: school absenteeism, influenza surveillance in the community, and comparison between school absenteeism and influenza surveillance in community. Each mentioned variable below was a column in the standard form. For school absenteeism, the number of schools, the school size, the grade of students, methods used for school absenteeism surveillance, the types of absenteeism (all-cause, ILI-related, or illness-specific), illness ascertainment methods for illness-related absenteeism, and the treatment of holiday in the analysis were extracted. For influenza surveillance in the community, the extracted data included the types of surveillance (influenza-like illness with or without laboratory test) and the information of laboratory surveillance (influenza strain and the use of test-positive number or proportion). For comparison between school absenteeism and influenza surveillance in the community, information included methods, period and lag time (day/week) of comparison, and estimated correlation coefficients.

### Data Analysis

For studies reporting regression coefficients, we used the approach by Rodgers and Nicewander [25] to transform them to Pearson correlation coefficient. We conducted random effects meta-analyses using the inverse variance method and restricted maximum likelihood estimator for heterogeneity to obtain the pooled correlation between school absenteeism and community surveillance [26-29]. Cochran *Q* test and the *I^2^* statistic were used to identify and quantify heterogeneity among included studies [30]. An *I^2^* value of more than 75% indicated high heterogeneity [31]. We conducted subgroup meta-analyses by the types of school absenteeism surveillance (all-cause vs ILI-related vs illness-specific), by the type of community surveillance (ILI vs lab-confirmed), with or without considering the delay effect of school absenteeism on community influenza. Meta-analysis on subgroups was only performed when there were at least 5 estimates. However, it should be noted that as *I^2^* could be biased in small studies, we only quoted the *I^2^* estimate when the number of studies was at least 10 [32]. We also conducted metaregressions to explore the impact of the following factors: types of school absenteeism, types of community surveillance, primary school or lower grade in school absenteeism surveillance, use of count data in school absenteeism surveillance, use of weekly data, and study type (prospective vs retrospective) in the analysis.

For studies using qualitative methods, in which only plots or tables of time series of school absenteeism and influenza activity in the community were provided without using any statistical comparison such as estimating correlations, a qualitative description of the relationship between these 2 surveillance systems was extracted. The used terms for the relationship in those articles was summarized. For the studies attempting to predict community surveillance by using school absenteeism, the following information about prediction was extracted: the types of data and its time period used for training for the models for prediction, data type and prediction methods, the type of data for validating the developed models and its time period, and the evaluation method.

## Results

### Overview

**Figure 1:**
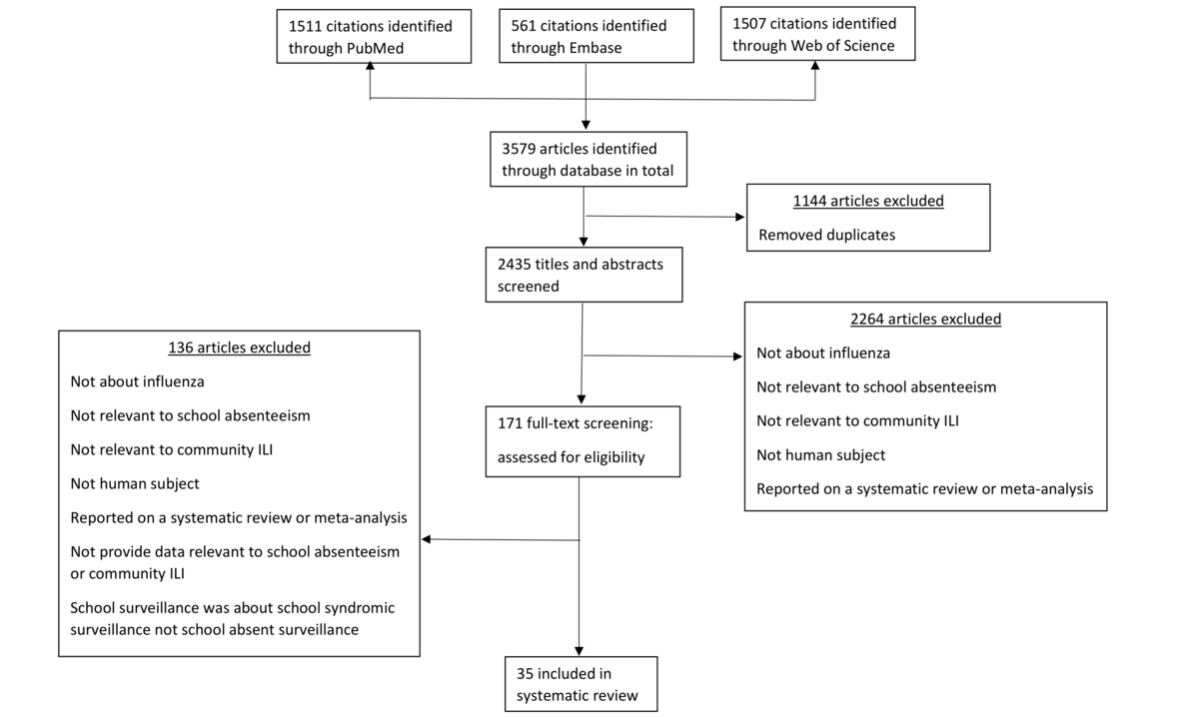
Process of systematic review. ILI: influenza-like illness.

In the systematic review, we identified 3579 studies in our search, and 1144 duplicated articles were excluded (Figure 1). After screening the titles and the abstracts of the remaining articles, we identified 171 studies that may contain relevant information for full-text screening. Among these, 35 articles (Table S1 in Multimedia Appendix 1) met the inclusion criteria and were included in this review [15-19,33-62], of which 9 [19,33,37,41,46,48,54,61,62] and 6 [17,18,40,42,51,53] were prospective and retrospective studies, respectively. In terms of school absenteeism surveillance, 22 [15,16,34-39,41,47-49,​^51^,^53^-^57^,^59^-^62^], 12 [15,17-19,35-37,44,45,50,54,61], and 8 [33,40,42,43,46,52,58,61] of the studies provided counts on all-cause, ILI-related, and illness-specific absenteeism, respectively. In terms of community surveillance, 25 [16-19,33-37,39-45,47,49-51,55,58-60,62] and 26 [15-17,19,33,34,36-43,46-49,52-54,56,57,59-61] studies provided the number of ILI cases and laboratory-confirmed cases, respectively. Moreover, 24 [15,17,19,33,35-38,40-45,​^47^-^49^,^51^,^54^,^56^-^58^,^60^,^62^] and 11 [16,18,34,39,46,50,52,​^53^,^55^,^59^,^61^] studies used weekly and daily scale of time series for analysis, respectively. In total, 7060 (we excluded 5 studies that did not provide this information) schools were included in our study. Of these, 2 articles [16,56] did not provide any comparison between school absenteeism and community surveillance; 16 articles [17-19,33,37,40-42,46,48,​^51^,^53^-^55^,^61^,^62^] with 7703 school years performed quantitative analysis by using statistical method to compute the correlation between school absenteeism and community surveillance; and 19 articles [15,16,34-36,38,39,43-45,47,49,50,52,56-60] with 2123 school years performed qualitative analysis comparing their trends visually by figures or plots. In addition, 6 [17,18,42,46,54,61], 5 [17,18,42,46,61], and 1 [37] articles considered time lag and reported comparisons of school absenteeism and community surveillance with 1-week, 2-week, and 3-week lags, respectively. Moreover, 25 studies excluded holidays in the comparison, while 10 studies did not provide any information. Among the 25 studies that excluded holidays in the comparison, 15 of them [16-18,33,37,45,48-53,55,61,62] used weekly absolute count in school absenteeism surveillance, which may not have accounted for the reduction in denominator due to holidays within the week. Overall, among 16 and 19 studies using quantitative and qualitative analysis, 15 and 17 studies supported the association between school absenteeism and community surveillance, respectively (Table 1).

**Table 1:** Summary of included studies for pooled analysis (n=35).

Characteristics				Studies, n		Reference
**Methods to assess correlation**						
	Quantitative analysis			16		N/A^a^
	Regression			4		[33,41,51,62]
	Correlation			11		[17-19,37,40,42,46,48,53,54,61]
	Other measures			1		[55]
	Qualitative analysis by comparing of trends in plots of time series			19		[15,16,34-36,38,39,43-45,47,49,50,52,56-60]
**Time lag between school absent and surveillance**						
	0 week			14		[17-19,33,40-42,46,48,51,53,54,61,62]
	1 week			6		[17,18,42,46,54,61]
	Others			1		[37]
**Association between school absent and surveillance**						
	**By quantitative analysis**					
		Significance for 0-week lag	6		[17-19,33,40,46]	
		Significance for 1-week lag	2		[17,18]	
		Significance for 2-week lag	2		[17,18]	
		Significance for 3-week lag	1		[37]	
		Unknown significance	8		[41,42,51,53-55,61,62]	
		No significant association	1		[48]	
	**By quantitative analysis**					
		With association	17		[16,34-36,38,39,43-45,47,49,50,56-60]	
		No detected association	2		[15,52]	

^a^N/A: not applicable.

### Correlation Between School Absenteeism and Community Surveillance

**Figure 2:**
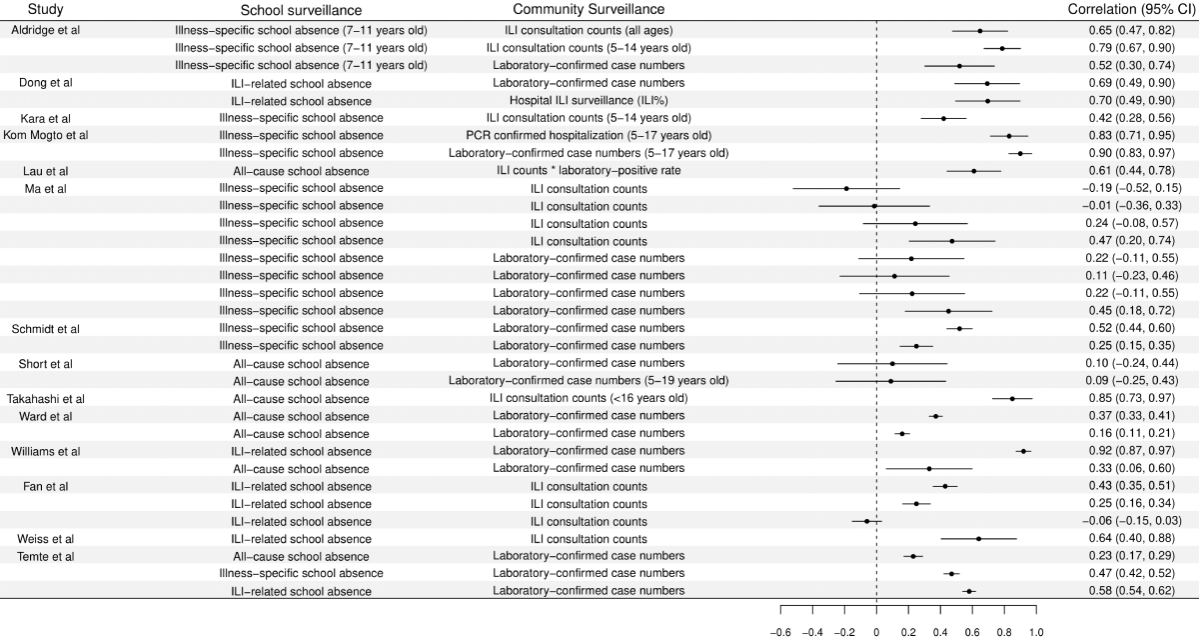
The temporal correlation between school absenteeism and influenza activity in community from identified studies. ILI: influenza-like illness; PCR: Polymerase Chain Reaction.

**Figure 3:**
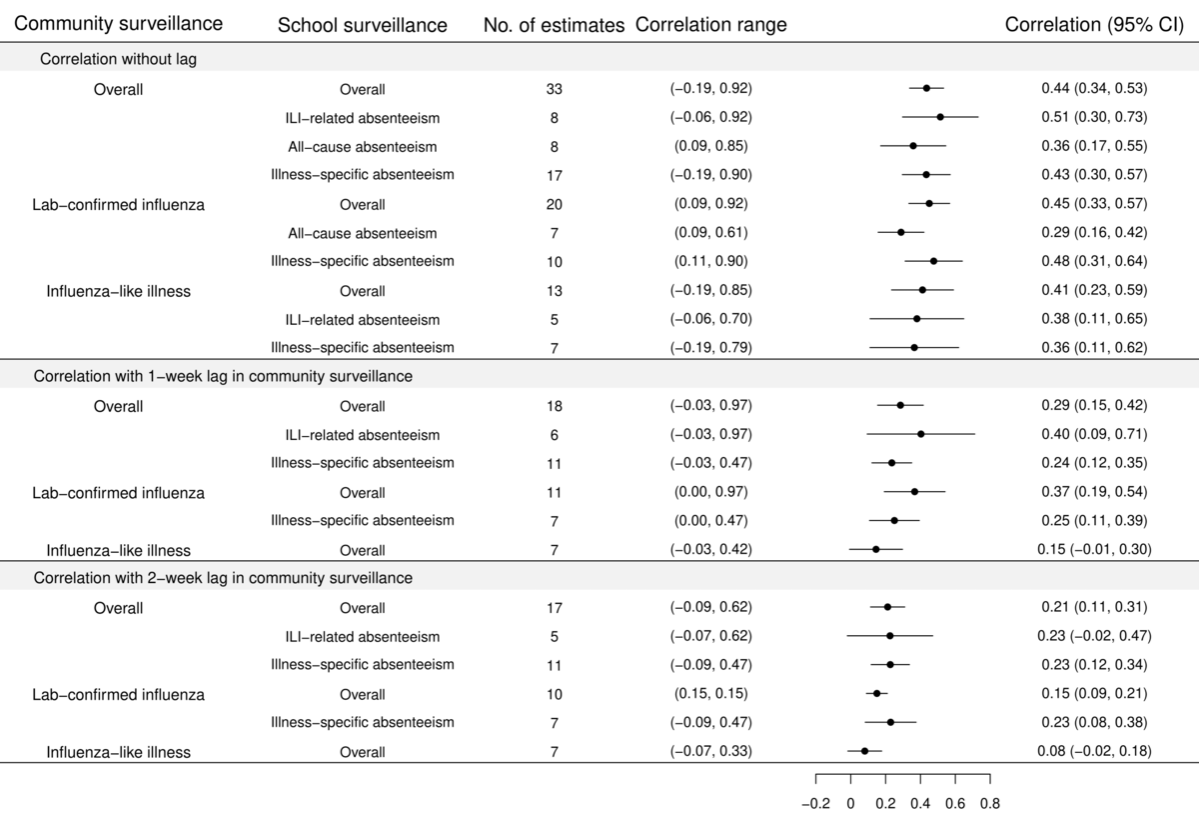
The pooled estimate of temporal correlations between school absenteeism and influenza activity in the community by types of school absenteeism surveillance and types of surveillance of influenza activity in community. ILI: influenza-like illness.

For quantitative analysis, all studies used regression coefficient or Pearson or Spearman correlation coefficient for evaluating the relationship between school absenteeism and community surveillance, except 1 [63], which used receiver operating characteristic to determine the accuracy of using school absenteeism to predict the occurrence of influenza outbreak, which was not included in our calculation of pooled correlation. Overall, 14 studies [17-19,33,40-42,46,48,51,53,54] provided 33 correlation estimates (Figure 2 [17-19,33,40-42,​^46^,^48^,^51^,^53^,^54^,^61^,^62^] and Table S2 in Multimedia Appendix 1), and 30 estimates supported a positive correlation between school absenteeism and community surveillance [18,42]. The correlation (without lag) between school absences and community surveillance was 0.44 (95% CI 0.34, 0.53), with high heterogeneity (Figure 3). In terms of community surveillance, the correlation between school absences and community surveillance using laboratory-confirmed cases (correlation: 0.45; 95% CI 0.33, 0.57) was higher than using the number of ILI (correlation: 0.41; 95% CI 0.23, 0.59), which was less specific. In terms of school absenteeism, the correlation between community and ILI-specific absenteeism (correlation: 0.51; 95% CI 0.30, 0.73) and illness-specific absenteeism (correlation: 0.43; 95% CI 0.30, 0.57) was higher than all-cause absenteeism (correlation: 0.36; 95% CI 0.11, 0.62). While there was a positive relationship between school absenteeism and the community surveillance with 1-week (correlation: 0.29; 95% CI 0.15, 0.42) and 2-week lag (correlation: 0.21; 95% CI 0.11, 0.31), the positive correlation was smaller compared with those without lag.

In the metaregression (Table 2), we estimated that the correlation between school absenteeism and the community surveillance with 1-week and 2-week lag was 0.19 (95% CI 0.04, 0.34) and 0.26 (95% CI 0.10, 0.41) lower than without lag. We found that the correlation between community surveillance and ILI-related absenteeism was 0.25 (95% CI 0.04, 0.45) higher than that between community surveillance and all-cause absenteeism. The correlation between school absenteeism and laboratory-confirmed community surveillance was 0.17 (95% CI 0.04, 0.30) higher than that between school absenteeism and ILI surveillance. We found that the correlation between school absenteeism and community surveillance from prospective studies was 0.19 (95% CI 0.05, 0.33) higher than from retrospective studies.

**Table 2:** Factors affecting the correlation between school absenteeism and community surveillance by metaregression.

Variables			No lag (95% CI)		1-week lag (95% CI)		2-week lag (95% CI)		Overall (0- to 2-week lag; 95% CI)
**School absence type**									
	All-cause absenteeism	Reference		Reference		Reference		Reference	
	ILI^a^-related absenteeism	0.20 (–0.11, 0.50)		0.39 (–0.11, 0.89)		0.21 (–0.16, 0.57)		0.25 (0.04, 0.45)	
	Illness-specific absenteeism	0.10 (–0.16, 0.35)		0.16 (–0.32, 0.64)		0.14 (–0.20, 0.47)		0.12 (–0.07, 0.30)	
**Community surveillance type**									
	ILI	Reference		Reference		Reference		Reference	
	Laboratory confirmed	0.08 (–0.14, 0.30)		0.30 (0.05, 0.54)		0.23 (0.03, 0.43)		0.17 (0.04, 0.30)	
**Study type**									
	Retrospective study	Reference		Reference		Reference		Reference	
	Prospective study	0.25 (0.04, 0.45)		0.12 (–0.20, 0.44)		0.07 (–0.19, 0.33)		0.19 (0.05, 0.33)	
**Lag^b^**									
	No lag	N/A^c^		N/A		N/A		Reference	
	1-week lag	N/A		N/A		N/A		–0.19 (–0.34, –0.04)	
	2-week lag	N/A		N/A		N/A		–0.26 (–0.41, –0.10)	
Only primary school and lower grade in school surveillance			–0.10 (–0.53, 0.34)		–0.21 (–0.59, 0.16)		–0.15 (–0.41, 0.11)		–0.14 (–0.36, 0.08)
Using count in school absenteeism (Reference: using rate or proportion)			0.03 (–0.23, 0.29)		–0.16 (–0.50, 0.18)		0.22 (–0.10, 0.54)		0.01 (–0.16, 0.18)
Using weekly scale (Reference: daily scale)			0.11 (–0.15, 0.38)		0.09 (–0.22, 0.41)		–0.07 (–0.33, 0.19)		0.06 (–0.10, 0.22)

^a^ILI: influenza-like illness.

^b^Number of weeks that school absenteeism data were leading community surveillance.

^c^N/A: not applicable.

### Qualitative Analysis for the Relationship School Absenteeism and Community Surveillance

In the qualitative analysis, there were 19 articles that reported the relationship between school absenteeism and community surveillance by presenting the time series of these surveillances (Table 3). Of these 19 studies, 2 [16,56] did not compare the relationship in plots of time series. Overall, a similar pattern was reported within school absenteeism and community surveillance in all studies (Table 3), except 2, which reported no detected association [15,52]. Moreover, 6 studies [35,36,43,47,49,50] compared the peak of school absent and peak of epidemics. Among these, 4 [35,36,43,50], 1 [49], and 1 [47] studies found that school absenteeism peaked before, at the same time, and after the peak of community surveillance, respectively. In addition, 15 studies concluded that school absenteeism was in concordance with, coincided with, or was associated with community surveillance, using terms including “trends are coincident” [34,47,59], “associated with” [16,34,36,56,57,60], “concordance with” [38,43,58], “similar trend” [39,44,50], or ‘’mirror” [45].

**Table 3:** Summary of term count for qualitative results.

Term combined and term used				Study		Comparison		Count		
**School absenteeism is in concordance with, coincided with, or is associated with community ILI^a^**										16
	Increases in absences coincided with community-wide ILI outbreaks			Besculides [34], 2005		All-cause absenteeism vs community influenza isolates		1		
	The second wave of school absenteeism coincided with the second round of community ILI			Schoub [47], 1994		All-cause absenteeism vs laboratory isolations		1		
	The drop in school absenteeism coincided with the epidemic			McCormick [59], 2010		All-cause absenteeism vs community ILI surveillance or virus-confirmed influenza		1		
	**School absenteeism is associated with community ILI**								6	
		The extent of school absenteeism is associated with the extent of community ILI peak	Besculides [34], 2005		All-cause absenteeism vs community influenza isolates					
		School absenteeism is associated with community ILI	Cheng [36], 2012		ILI-related absenteeism vs laboratory isolations					
		Influenza activity was reflected by the school absenteeism rates	Chin [57], 1974		All-cause absenteeism vs laboratory-confirmed influenza					
		Influenza activity was associated with or reflected by school absenteeism	Rubin [60], 1975		All-cause absenteeism vs community ILI surveillance					
		School absenteeism was less timely than laboratory data	Chu [16], 2013		All-cause absenteeism vs laboratory-confirmed influenza					
		Close temporal correlation between school absenteeism and the isolation of strains of influenza virus	Olson [56], 1980		All-cause absenteeism vs laboratory-confirmed influenza					
	**School absenteeism is concordant with community ILI**								3	
				Jaeger [38], 2011		All-cause absenteeism vs laboratory-confirmed influenza				
				Mook [43], 2007		Illness-defined absenteeism vs community ILI surveillance				
				Lenaway [58], 1995		ILI-related absenteeism vs community ILI surveillance				
	**Similar trend**								3	
				Janusz [39], 2011		All-cause absenteeism vs community ILI surveillance				
				Nasrullah [44], 2012		ILI-related absenteeism vs community ILI surveillance				
				Suzue [50], 2012		ILI-related absenteeism vs community ILI surveillance				
	School absenteeism mirrored or corresponded to community ILI			Read [45], 2021		Influenza-confirmed absenteeism vs community virus–confirmed influenza		1		
**Comparison by peak time**										6
	Peak at the same time			Sigmundsdottir [49], 2010		All-cause absenteeism vs laboratory confirmed cases		1		
	**School absenteeism peak preceded or was ahead of the epidemic peak**								4	
				Bollaerts [35], 2010		All-cause absenteeism vs community ILI surveillance				
				Cheng [36], 2012		ILI-related absenteeism vs laboratory isolations				
				Mook [43], 2007		Illness-defined absenteeism vs community ILI surveillance or laboratory isolations				
				Suzue [50], 2012		ILI-related absenteeism vs community ILI surveillance				
	Virus isolation commence and peak before the school absenteeism			Schoub [47], 1994		All-cause absenteeism vs laboratory isolations		1		
**No detected association**										2
	No related outbreaks were detected, and no peaks were found			Tan [52], 2014		ILI-related absenteeism vs laboratory-confirmed cases		1		
	Within seasons, cases peaked in winter, whereas county-level absences varied throughout the year			Quandelacy [15], 2021		ILI-related absenteeism or all-cause absenteeism vs community virus–confirmed influenza		1		

^a^ILI: influenza-like illness.

### Prediction of Community Surveillance From School Surveillance

Six studies attempted to predict community ILI from school absenteeism (Table S3 in Multimedia Appendix 1), 3 of which [15,41,62] predicted the influenza rate (case number), and 3 [18,53,55] predicted the occurrence of outbreaks. All the studies agreed that school absenteeism surveillance was of good use for influenza outbreak detection [15,18,41,53,62], except 1 using all-cause absenteeism [55]. Among these, 4 studies [15,53,55,62] and 2 studies [18,41] used the all-cause absenteeism and ILI-related absenteeism for prediction, respectively. Moreover, 1 [18] and 5 [15,41,53,55,62] studies used mechanistic models (SEIR) and statistical models, respectively, 3 of which [41,53,62] considered the autocorrelation of the time series. In term of evaluation, those studies that predicted the occurrence of outbreaks used receiver operating characteristic curves [18,55] and false-positive rate (1 minus sensitivity) [53]. For those studies that predicted the number of cases, 1 used the model fit [41], 1 used mean absolute error [15], and 1 used Akaike Information Criterion value [62]. Two articles [15,53] conducted out-sample testing to evaluate the performance.

## Discussion

### Principal Findings

In this study, we summarized the practice of school absenteeism surveillance and their potential use on monitoring and predicting influenza activity in the community. Studies could broadly be classified as quantitative and qualitative. In quantitative studies, most studies used temporal correlation to quantify the relationship between school absenteeism and influenza activity in the community. In qualitative studies, time series between these 2 data streams were compared visually. Overall, we found a moderate correlation between school absenteeism and influenza activity in the community based on quantitative studies. This suggested the potential to use school absenteeism data to monitor or predict influenza activity in the community.

The measure of school absence could be classified to nonspecific (all-cause absent) and specific (illness- or ILI-specific absence). We found that ILI-specific absenteeism had a higher correlation with the community surveillance, compared with all-cause absenteeism, which was consistent with the findings from another review [64]. While using specific measure of school absenteeism could be slightly more accurate [54], the implementation of using more specific absenteeism surveillance in schools should be considered jointly with the associated costs. Those specific measures may require more resources to obtain, such as follow-up of the reasons of absence, which may also jeopardize the timeliness of the school absence data. Moreover, the willingness of school participation may be lower due to the higher requirement of resource; hence the sample size may decrease. Such factors should be considered when deciding the use of all-cause absence or ILI-specific absence to monitor influenza activity. The actual cost of implementation of using more specific absenteeism surveillance in schools was likely different by regions and countries; therefore, our studies could not provide recommendation on this.

A number of studies proposed that one of the values of school absenteeism surveillance was its lead on the traditional surveillance [35,36,43,50]. However, we found that the correlation between school absenteeism and community surveillance without lag was the highest. In addition, the correlation between school absenteeism and 1-week lag of community surveillance was only marginally smaller than community surveillance without lag. Hence, the school absenteeism surveillance could at most lead influenza outbreaks by 1 week. Therefore, despite the moderate correlation between school absenteeism and community surveillance, the use of school absenteeism as a surveillance tool may require further exploration and development of methodology. In particular, we found that the correlations between school absenteeism and community surveillance from prospective studies were higher than from retrospective studies, suggesting that prospective data collection could improve the accuracy by carefully checking the data with timely correction.

More than half of the studies only use qualitative approaches to explore the relationship between school absenteeism and influenza activity in the community, in which only figures of time series of these 2 surveillances were compared visually. Such approaches were relatively subjective, and further quantitative comparison should be performed. Furthermore, almost all quantitative comparisons were based on temporal correlation, which required further steps to determine the usefulness of school absenteeism for monitoring or predicting influenza activity in the community, such as the development of prediction or forecasting tools.

Only 6 out of 35 studies attempted to develop methods to use the school absenteeism data to predicted influenza activity in the community. However, those applied approaches were suboptimal compared with other influenza-forecasting approaches [65,66]; particularly, the potential of more advanced statistical approaches was less explored, as well as the lack of validation. In terms of modeling fitting, only 2 out of 6 studies conducted out-sample evaluation on their forecasting approach to avoid overfitting. In terms of model evaluation, all studies evaluated the point forecast, but none of them evaluated the prediction intervals from those models. The evaluation of interval forecast by some proper score rules would also be important to use the prediction performance [66,67] and to support the accuracy of the predictions. In future studies, using school absenteeism data in more well-developed models should be explored, including mechanistic models (ie, SIR-type) [65,66], and statistical models, such as generalized additive model and random forecast regression, which could particularly handle nonlinear relationship [68].

### Limitations

Our study may have some limitations. First, we did not summarize the change of rule on the school attending in the identified studies. For example, many regions may not allow students to attend school when they have fever, particularly during or after the 2009 pandemic influenza outbreaks. Therefore, our study may not be able to evaluate this impact on the relationship between school absenteeism and community surveillance. Second, we did not stratify the analysis by type of reported correlation coefficients (Pearson or Spearman). However, we expected that the direction of correlation should be the same. Third, most studies did not report information of burden or costs of implementing school surveillance. Therefore, it was impossible for us to determine the real-life impact or cost-effectiveness of different types of surveillance. Finally, we could not rule out other potential cofounders in the identified relationship between school absenteeism and influenza activity in the community. For example, information of school or class size was not available.

### Conclusion

We found there was a moderate correlation between school absenteeism and influenza activity in the community. We found that the correlation between influenza activity and ILI-specific absenteeism was higher than all-cause absenteeism. However, implementing more specific surveillance in school may require careful consideration, since more resources may be required, and it may have a negative impact on the willingness of school participation. There was potential for using school absenteeism as a surveillance and prediction tool of influenza activity. A further development of methodology was required to use and optimize such usage. In particular, more statistical models should be explored, and the validation of prediction performance is missed in most studies.

## Appendix

Multimedia Appendix 1 Supplementary tables.

## Abbreviations

ILI: influenza-like illness
PRISMA: Preferred Reporting Items for Systematic Review and Meta-analysis
