# Supplementary material for: Monitoring School Absenteeism for Influenza-Like Illness Surveillance: Systematic Review and Meta-analysis
Source: JMIR Public Health Surveill. 2023 Jan 11;9:e41329. doi: 10.2196/41329 (PMC9878370; doi:10.2196/41329)
Supplement: Multimedia Appendix 1 [file publichealth_v9i1e41329_app1.docx]

**Table S1.** Summary of identified studies.

| ***Author (year)*** | ***Location*** | ***Study period*** | ***Study type*** | ***Grade of student*** | ***Measure of school absenteeism*** | ***Exclusion of days in analysis*** | ***Measure of community surveillance*** | ***Method of assessing relationship*** |
| --- | --- | --- | --- | --- | --- | --- | --- | --- |
| Aldridge (2016) | England | 2011/12 and 2012/13 | Prospective study | Secondary schools | Weekly total number of half-days illness-related absences (first year), prevalence of daily illness-related school absence (second year) | School holidays such as Christmas or weekends | Royal College of General Practitioners (RCGP) influenza like illness (ILI) episode incidence rate per 100,000 population | Univariable linear regression |
| Besculides (2005) | New York City, US | 2001/02, 2002/03, and 2003/04 | Retrospective study | Primary* and secondary* school, High School | Daily all-caused percent absent | Holiday or abnormal date such as Halloween | 1) the daily number of emergency department (ED) patients ages 5–17 complaining of fever or influenza-like illness from an existing ED surveillance system  2) the weekly number of influenza A and B isolates identified at NYC reference laboratories | Time trends** |
| Bollaerts (2010) | Belgium | Week 28-week 52 in 2009 | Retrospective study | Nursery, primary and secondary schools | Weekly ILI-specific and all-cause school absenteeism rates | NA | weekly ILI incidence (per 100,000 inhabitants) | Time trends** |
| Cheng (2012) | Hong Kong | March 2008–June 2011 | Prospective study | Primary and secondary schools | Weekly ILI-specific and all-cause absenteeism rates | School holidays or school closure | 1) Weekly ILI (defined as fever plus cough or sore throat)  consultation rates in sentinel networks of outpatient clinics in the private sector  2) Proportion of influenza A and B virus isolations among all specimens  submitted to the reference laboratory for Hong Kong Island at Queen Mary Hospital | Time trends** |
| Chu (2013) | Ontario, Canada | 1 April 2009 - 31 January 2010 | Retrospective study | Primary* school (kindergarten to grade eight) | Daily all-cause absenteeism rates and counts | Weekends, statutory holidays and professional development days | Emergency department (ED) visits, laboratory-confirmed A(H1N1) cases | Timeliness^ |
| Dong (2017) | Tianjin, China | 30 September 2013 - 30 March 2014 | Retrospective study | Primary and secondary schools | Daily counts of ILI cases and weekly ILI-specific absences | Holiday breaks (e.g. national holiday) | 1) influenza virus positive rates (lab).  2) The number of ILI cases and total number of clinical consultations | Spearman correlation analysis |
| Ip (2017) | Hong Kong | September 2012 - June 2014 | Prospective study | Primary and secondary schools | Weekly all-cause and ILI-specific absentees count | Abnormal school days, school breaks and holidays | 1) the proportion of ILI consultations among general outpatient attendance in the existing sentinel GP surveillance network  2) positive influenza detection rate from hospital laboratory samples | Timeliness^, Cross-correlation coefficient (CCC) analyses |
| Jaeger (2011) | Ontario, Canada | 1 September 2009 - 1 December 2009 | Retrospective study? | Primary and secondary school | Local primary and secondary school weekly all-cause absenteeism rates | NA | laboratory confirmed H1N1/ influenza A cases | Time trends** |
| Janusz (2011) | Chicago, Illinois, US | 13 April 2009 - 17 May 2009 | Prospective study | Primary* schools | The proportion of daily all-cause absent students (absenteeism) | NA | Outpatient visits for ILI and influenza testing | Time trends** |
| Kara (2012) | Birmingham, UK | September 2006 - September 2009 | Retrospective study | Primary and secondary | Mean weekly rate of term-time absenteeism (illness-related) | Holidays and weekends | Weekly incidence rates of ILI/100 000 population, the number of samples positive for influenza A(H1N1) | Correlation, time trends** |
| Kom Mogto (2012) | Quebec, Canada | 2 October 2009 – 12 December 2009 | Prospective study | Primary* and high schools | Weekly incidence, weekly prevalence and cumulative incidence of schools reporting an absenteeism rate >10% (ILI-specific) | NA | Provincial surveillance for laboratory-confirmed cases and hospitalizations due to pandemic influenza | Non-parametric Spearman coefficient |
| Lau (2012) | Hong Kong | 2004 –2009 | Prospective study | NA | The proportion of weekly all-cause students absent | School holidays, such as summer holidays | The product of the laboratory influenza detection rate and the GP ILI consultation rate | Multivariate dynamic linear time series model |
| Ma (2015) | Sweden | 2009/10 -2012/13 | Retrospective study | From primary* schools to high schools | Weekly illness-related school absences | School holidays | The proportion of patients with ILI per 100 000 patients registered with the practice, the number of laboratory-confirmed cases of influenza | Pearson’s correlation statistic |
| Mook (2007) | England | 14 January 2007 - 20 May 2007 | Prospective study | Primary and secondary schools | Weekly illness-related absenteeism | Holidays | RCGP ILI episode incidence rate (all age/ 5-14y), the number of positive influenza samples | Time trends** |
| Nasrullah (2012) | Georgia, US | 3 August 2008 –13 November 2008 | Prospective study | K–12 schools | Significant respiratory illness and absenteeism (SRIA)(weekly/ILI-specific) | NA | proportion of influenza-like illness (ILI) in the emergency departments | Time trends** |
| Olson (1980) | Taipei, Taiwan | December 1975 - February 1976 | Retrospective study | grades 1-6 and 9-12 | Weekly all-cause absenteeism rates | NA | Throat swabs sample of Febrile outpatients with upper respiratory  illnesses | Time trends** |
| Quandelacy (2021) | Allegheny County, Pennsylvania, US | 2007/08 and 2010-2015 | Prospective study | Primary and secondary schools (K-12 grades) and accelerated academies and charter schools | Weekly all-cause absences and school enrollment data | School closures (e.g., spring break, federal holidays,  weekends) | Virologic confirmed influenza case data | Negative binomial models |
| Read (2021) | Pittsburgh, Pennsylvania, US | 2012/13 | Prospective study | Kindergarten - grade 12 | Weekly ILI–specific absences + swab (conduct virus test for the ill absent schoolchildren) | NA | Weekly influenza cases | Time trends** |
| Schmidt (2010) | London, UK | 2005/06, 2006/07 | Prospective study | Primary schools (years 1- 6,4schools, years 1-2, 2 schools) | Daily prevalence/ incidence of illness-related absence | Weekends and school holidays | Laboratory reports of confirmed influenza A and B cases | descriptive statistics, and simple graphical descriptive analysis, cross-correlation analysis (lag) |
| Schoub (1994) | Witwatersrand, South Africa | 1992-1993 | Prospective study | Primary schools and high schools | Weekly all-cause absenteeism rate | NA | Virus isolation, acute respiratory morbidity index (ARMI) | Time trends** |
| Short (2011) | Pennsylvania, US | 25 September 2009 – 4 June 2010 | Prospective study | Grades K-grades 12 | The weekly number of students all-cause absent from school,  the number of students seen in school for ILI by the school nurse or a representative | Weekends or school breaks | Laboratory-confirmed influenza cases | Time trends**, Spearman correlation |
| Sigmundsdottir (2010) | Iceland | 29 June 2009 - 27 December 2009 | Retrospective study | primary* schools / 6–15 y | The weekly number of days all-cause absent | NA | The number of reported ILI cases, number of respiratory samples and proportion positive | Time trends** |
| Suzue (2012) | Takamatsu city, Japan | 13 October 2009 - 12 January 2010 | Prospective study | Kindergartens, primary* schools, secondary* schools and high schools / 3–18 y | SARSID (the daily number of ILI-specific absenteeism) | Outside five working days per week | NESID (the number of cases of influenza for five working days per week/ Numbers of patients  with influenza who visited the sentinel clinics or hospitals in a week) | Join-point regression program, parallelism and the coincidence |
| Takahashi (2001) | Japan | Week 2 - week 11 of 97/98 season and 98/99 season | Retrospective study | 4-15y | Weekly number of all-cause absent children per 1000000 | NA | average number of children with ILI under 16y per sentinel site | Regression |
| Tan (2014) | Hubei Province, China | 19 September 2011 -31 December 2011 | Prospective study | Primary* schools (years 1-6) | Daily number of illness-related absence records | Holiday and weekends | influenza cases | Time trends** |
| Ward (2019) | Wellington-Dufferin-Guelph, Ontario, Canada | September 2008 - June 2018, not including the 2009/  10 school year | Retrospective study | Primary and secondary schools | Daily number of all-cause absent | Weekends, statutory and school board holidays, and school breaks (for example, winter holidays, March break, and the summer holidays) | laboratory-confirmed influenza cases | Spearman correlation, Cross-correlation  up to 15 lags |
| Williams (2013) | Denver Metropolitan Region, Colorado, US | 31 August 2009 - 25 June 2010 | Prospective study | Prekindergarten through grade 12 students | Weekly rate of all-cause absenteeism and ILI-related absenteeism | Weekends, holidays, and summer breaks | weekly counts of reported laboratory-based confirmed cases of 2009 H1N1 among hospitalized patients | Pearson r |
| Egger (2012) | New York City, US | 6 September 2005 - 26 June 2009 | Retrospective study | Primary*, secondary* schools | Daily all-cause school absenteeism | Non-school days | Daily school nurse visits for fever/influenza syndrome | Absenteeism z-score time series (for best lag days predict) |
| Fan (2014) | Hubei Province, China | 1 April 2012 – 30 June 2013 | Retrospective study | Primary school | The daily number of ILI-specific absent students | Weekends and vacations | The daily count of patients with symptoms of influenza-like illness | Spearman’s rank correlation |
| Chin (1974) | California, US | 1968 - 1973 | Retrospective study | NA | Weekly percentage of all-cause school absent | Non-school days | laboratory-confirmed influenza cases | Time trends |
| Lenaway (1995) | Boulder, US | 1988/89 – 1992/93 | Prospective study | Primary*, secondary*, and high schools | weekly illness-related absent average rate  exceeds 7.5 percent of the current school census | Non-school days | weekly reported  number of medically attended influenza-like illness cases | Time trends |
| Rubin (1975) | US | November -March 1972/73, November - March 1973/74 | Prospective study | NA | Weekly percentage of all-cause school absent | Christmas vacation | weekly number of emergency room visits | Time trends |
| Weiss (2019) | Atlanta, Georgia, US | 29 September 2013 - 22 March 2014 | Prospective study | Secondary* school | Weekly number of school all-cause absent | Thanksgiving and winter breaks | Weekly number of statewide influenza-like Illness cases | Regression |
| McCormick (2010) | Cameron County, US | 27 April 2009 - 15 May 2009 | Prospective study | Primary* and secondary* school, high school, child daycare facilities | Daily average percent of all-cause school attendance | NA | Daily number of influenza-like Illness cases, daily number of laboratory-confirmed influenza A cases | Time trends |
| Temte (2022) | Dane County, Wisconsin, US | 2 September 2014 - 13 March 2020 | Prospective study | Primary* schools secondary* school, high school | Daily counts of all-cause, illness-related and ILI-specific absent students | Weekend days, vacation days, school closure days, or during the summer | Weekly number of medically-attended laboratory-confirmed influenza cases | Cross-correlation  up to 15 days lag |

*primary = elementary in US, Canada, Sweden, Iceland and China

Secondary = middle school in US; Secondary = junior high school in Japan

** comparison in the trend in time series between school absenteeism surveillance and influenza surveillance

^ comparison in the time between syndromic surveillance alert and the laboratory alert date

**Table S2.** Summary of identified studies with using qualitative methods.

| ***Author (year)*** | ***Sample size*** | ***Items for comparison*** | ***Comparing items*** | ***Description of relationship between school absent and community surveillance based on the article*** | ***Conclusion of relationship between school absent and community surveillance*** |
| --- | --- | --- | --- | --- | --- |
| Besculides (2005) | 118*3 days (2001–02, 2002-03, 2003-04 school year) | fig 2, 3, 4 | Adjusted daily percent absent among elementary/middle school and high school students vs Emergency Department visits for fever  and flu-like illness among children age 5–17 / the number of influenza A and influenza B isolates identified | Increases in absences coincided with four community-wide outbreaks of influenza-like illness | School absent was correlated with community surveillance |
| Bollaerts (2010) | 17 weeks (36-52 week in 2009) | fig 1 | daily absenteeism rates for (1) nursery school, (2) primary school and (3) secondary school data by 3 community vs weekly influenza-like-illness (ILI) incidence per 100,000 inhabitants | The peaks of the absenteeism curves  preceded the epidemic peak at week 44 with ± 2 weeks | The peaks of the absenteeism curves  preceded the epidemic peak |
| Cheng (2012) | 118 weeks (March 2008–June 2011) | figure | Weekly overall/ILI school absenteeism vs Weekly ILI consultation rates /Proportion of influenza A and B virus isolations | Clear and sharp peaks  were detectable from both the overall and ILI-specific data during most of these influenza seasons which generally  occurred 1–3 weeks ahead of the peaks in the laboratory data | the peaks of overall and ILI-specific data occurred ahead of the peaks in the laboratory data |
| Chu (2013) | 304 days (1 April 2009 - 31 January 2010) | fig 2b | Comparisons in time between alert dates from laboratory and syndromic surveillance | school absenteeism was less timely than laboratory data | Alert from school absenteeism data was later than that of laboratory surveillance |
| Jaeger (2011) | 14 weeks (September 1 to December 1, 2009) | fig 1 | 5-day average of schools >10% absenteeism vs laboratory confirmed H1N1or influenza A cases | Figure 1 shows the parallel development of the peak of the wave and its subsidence | parallel development of the peak of the wave and its subsidence within school absent and community surveillance data |
| Janusz (2011) | 35 days (13 April to 17 May 2009) | fig 1 | Proportion of students’ absent vs Confirmed cases of pH1N1 / Number of ED and outpatient visits for ILI | Trends in surveillance data peaked during the same week and rapidly decreased to near baseline | Trends in school absent and confirmed pH1N1 cases peaked at the same week |
| Mook (2007) | 19 weeks (week 02- week 20 2007) | fig 2, fig 3 | absenteeism due to illness vs RCGP rate (all age/ 5-14y)/ influenza positives | [1] The peak RCGP rate and number of positive influenza samples was one week after peak week for illness-defined absenteeism in week 07/07, [2] School illness-defined absence data peaked during week ending 11/02/07, the same time as the RCGP rate for the 5-14 years age group | The peak of community surveillance was coincided with/ slightly after the peak of school absent |
| Nasrullah (2012) | 15 weeks (August 3–November 13, 2008) | figure 5 | Percentage of schools reporting significant respiratory illness and absenteeism (SRIA) vs proportion of influenza-like illness (ILI) in the emergency departments | there was a rapid  increase in the proportion of schools reporting SRIA during August 10–September 4, with a peak during the week of September 14–18. A similar pattern was observed over time in relation to the proportion of Georgia emergency department visits attributable to respiratory illness | similar patterns in the proportion of schools reporting SRIA and the proportion of Georgia emergency department visits attributable to respiratory illness |
| Olson (1980) | 39 weeks (December 1975 - February 1976) | Table 1, fig 1, 2 | excess absences among primary school children vs the isolation of strains of influenza  virus (primarily influenza B) | close temporal correlation between  excess absences among primary school children and the isolation of strains of influenza  virus (primarily influenza B) | School absent was correlated with community surveillance |
| Quandelacy (2021) | 213 weeks (2007-2008, and 2010-2015) | fig 1 | Weekly reported virologically confirmed influenza cases vs all-cause and influenza-like-illness (ILI) specific absences | Within seasons, cases peaked in the winter whereas county-level absences varied throughout the year. | NA |
| Read (2021) | 16 weeks (10 Dec 2012- 25 Mar 2013) | fig 1 | The number of virologic confirmed influenza virus type among absent school children vs Weekly influenza cases (stratified by influenza type) in the county | the pattern of influenza-associated absences confirmed during the study period mirror emergency department and outpatient provider-reported, virologically confirmed influenza from the Allegheny County Department of Health | the pattern of influenza-associated absences mirrors the community surveillance |
| Schoub (1994) | 30 weeks (Week 15-39, 1992 & week 15-39, 1993) | fig 1 | Actual absentee rate vs influenza isolations/ Acute respiratory morbidity index (ARMI) | [1] Virus isolation commenced before the rise in weekly absenteeism rate and the peak of virus isolation preceded the peak of absenteeism. [2] the second peak of absenteeism coincided with a second round with virus isolation | There is correlation with school absenteeism and the virus isolation |
| Sigmundsdottir (2010) | 20 weeks (week 33-week 52 2009) | Fig 1, fig 5, fig 6 | School absenteeism vs laboratory confirmed cases | [1] The number of samples and the percentage of samples positive increased in late September (week 40) and peaked in mid-October (week 42). [2] A sharp increase was  observed in October 2009, with a high peak in mid-October (week 42) | The increase and peak of influenza cases coincided with that of school absenteeism |
| Suzue (2012) | 52 days (Oct 13, 2009 - Jan 12, 2010) | fig 1, table 2 | SARSID (School  Absentees Reporting System for Infectious Disease) vs NESID (National Epidemiological Surveillance of infectious Diseases) | Similar trends were observed in SARSID and NESID | Similar trends were observed in school absent and community surveillance |
| Tan (2014) | 72 days (September 19, 2011 - December 31, 2011) | fig 3B | absent on fever vs influenza cases | no related outbreaks were detected by the system, and no peaks were found. | Limited information in school absent for community surveillance |
| Chin (1974) | 1056 days (October – May 1968-1973) | chart 1 | weekly school absenteeism vs laboratory confirmed influenza cases | In each instance, it was reflected by a  pronounced increase in school absenteeism rates | Influenza activity was reflected by the school absenteeism rates |
| Lenaway (1995) | 60 weeks (1988/89 -1992/93) | figure | the number of schools reporting excess absenteeism vs the weekly reported number of ILI cases | The overall pattern suggests concordance between the two measures for each outbreak | The overall pattern is concordant between school-based surveillance system and the preexisting sentinel surveillance system |
| Rubin (1975) | 11 weeks (8 December 1973 – 2 March 1974) | fig 7 | school absenteeism vs emergency room visits/influenza B activity | [1] School absenteeism was markedly increased during the influenza-B outbreak. [2] School absenteeism appeared to be a reliable index of influenza-B activity. [3] monitoring school absenteeism during epidemics of influenza-B was probably a reflection of the epidemiology of influenza-B | Influenza activity was associated with school absenteeism |
| McCormick (2010) | 14 days (April 27, 2009 to May 15, 2009) | fig2, 5 | school attendances vs ILI/ rapid influenza diagnostic test/laboratory results | The drop in school and daycare attendance that coincided with the epidemic | The drop in school absenteeism coincided with the epidemic |

**Table S3.** Summary for the studies using school absenteeism to predict community surveillance.

| ***Author (year)*** | ***Study period*** | ***Prediction target*** | ***Data for training/fitting the prediction models*** | ***Model for prediction*** | ***Period for training data*** | ***Testing data*** | ***Period for testing data*** | ***Method for evaluation*** | ***Overall conclusion*** |
| --- | --- | --- | --- | --- | --- | --- | --- | --- | --- |
| Egger (2012) | September 6, 2005 - June 26, 2009 | Occurrence of outbreak, defined as (z-score of daily count of visits for fever/influenza syndrome > 3) | Daily school district-level count of school nurse visits for fever/influenza syndrome, all-cause absenteeism data | Negative binomial regression models | September 25, 2006 - June 26, 2009 | In-sample | Same as training data | Receiver operating characteristic (ROC) curves for detecting an outbreak | Non–disease-specific absenteeism data alone are ***of little use*** for school-based influenza surveillance |
| Fan (2014) | April 1, 2012 - June 30, 2013 | Occurrence of outbreak (the number of current cases exceeds three deviations above the baseline mean) | ILI-related school absence surveillance | SEIR models & Healthcare-seeking behaviors model | April 1, 2012, - June 30, 2013 | In-sample | Same as training data | ROC curves and activity monitoring operation curves (AMOC) for validity and timeliness of syndromic surveillance | School absenteeism surveillance ***exhibited a satisfactory performance*** on outbreak detection |
| Lau (2012) | 2004–2009 | The level (low, medium and high) and trend (decreasing, stable, increasing) of influenza activity, inferred by the product of the laboratory influenza detection rate and the GP ILI consultation rate in the same week | ILI consultant and ILI-related school absenteeism | Multivariate dynamic linear time series model | 2004–2009 | In-sample | Same as training data | Univariate dynamic linear model/box-plot | The estimated influenza level/trend from the multivariate model showed **higher correlation with/ stably reflected** the change in influenza activity |
| Quandelacy (2021) | 2007-2008, & 2010-2015 | Weekly number of laboratory-confirmed influenza cases | Seasonal variables alone, and including weekly all-cause county-level school absences at one-, two-, and three-week lags | Negative binomial regression models | 2010-2015 | In-sample + out-of-sample | Same as training data | Mean absolute error (MAE) and relative mean absolute error (relMAE) | Including school absences in seasonal models ***improved*** community-level confirmed influenza predictions over multiple seasons |
| Ward (2019) | September 2008 -June 2018, excluding the 2009–2010 school year | Occurrence of outbreak (Exponentially weighted moving average statistic surpassed the upper limit or the predicted probability of at least one laboratory-confirmed case surpassed the defined probability threshold between 0.1 and 0.6) | A 10% all-cause absenteeism cut-off for the absenteeism averaged across certain schools | Mixed logistic regression models | September 2008 - June 2009 | Out-of-sample | September 2010 - June 2018 | False alarm rate (FAR) and accumulated days delay (ADD) | School absenteeism can be a ***useful tool*** for alerting public health to upcoming influenza epidemics |
| Weiss (2019) | 29 September 2013 - 22 March 2014 | the expected number of statewide influenza-like illness cases | the daily number of students who were signed out by their parent or guardian with the written reason of sign-out as “sick”, “doctor”, or “other” (all-cause) | first-order autoregressive model with  Poisson noise | 29 September 2013 - 22 March 2014 | In-sample | Same as training data | Akaike Information Criterion value | The surveillance  data collected from school sign-out logs  were **highly correlated with** the influenza like illness data, strongly suggesting the  possibility of detecting the onset of an outbreak sooner than current methodologies |
